# PIVKA-II combined with alpha-fetoprotein for the diagnostic value of hepatic tumors in children: a multicenter, prospective observational study

**DOI:** 10.1007/s12072-024-10668-4

**Published:** 2024-04-16

**Authors:** Hongxiang Gao, Chenjie Xie, Jing Wang, Ji Ma, Shijian Liu, Li Xie, Yijie Zheng, Rui Dong, Shan Wang, Yongjun Fang, Yurui Wu, Xianwei Zhang, Xianying Lu, Yang Li, Weisong Li, Qiuhui Pan, Min Xu, Song Gu

**Affiliations:** 1 Department of Pediatric General Surgery, Shanghai Children’s Medical Center, School of Medicine, Shanghai Jiao Tong University, Dongfang Road No. 1678, Pudong New District, Shanghai, 200127 China; 2 Department of Laboratory Medicine, Shanghai Children’s Medical Center, School of Medicine, Shanghai Jiao Tong University, Shanghai, 200127 China; 3 Child Health Advocacy Institute, Shanghai Children’s Medical Center, School of Medicine, Shanghai Jiao Tong University, Shanghai, 200127 China; 4 Clinical Research Institute, Shanghai Jiao Tong University School of Medicine, Shanghai, 200025 China; 5 Department of Medical Affairs, Wuxidiagnotics, Shanghai, 200131 China; 6 Department of Pediatric Surgery, Children’s Hospital of Fudan University, Shanghai, 201102 China; 7 Department of Pediatric Surgical Oncology, Children’s Hospital of Chongqing Medical University, Chongqing, 400014 China; 8 Department of Hematology and Oncology, Children’s Hospital of Nanjing Medical University, Nanjing, 210008 China; 9 Department of Minimally Invasive Surgery, Qilu Children’s Hospital of Shandong University, Jinan, 250022 Shandong China; 10 Department of Oncology Surgery, Children’s Hospital Affiliated to Zhengzhou University, Zhengzhou, 450018 China; 11 Department of Pediatric Surgery, Anhui Provincial Children’s Hospital, Hefei, 230051 China; 12 Department of Pediatric Hematology/Oncology, Sun Yat-Sen Memorial Hospital, Sun Yat-Sen University, Guangzhou, 519000 China; 13 Department of General Surgery, Pediatric Surgery, The First Affiliated Hospital of Anhui Medical University, Hefei, 230022 China

**Keywords:** Alpha-fetoprotein, Biomarker, Children, Diagnostic, Hemangioendothelioma, Hepatic tumor, Hepatoblastoma, Histopathology, Protein induced by vitamin K antagonist-II, Malignant tumor

## Abstract

**Background:**

To investigate whether protein induced by vitamin K antagonist-II (PIVKA-II) combined with alpha-fetoprotein (AFP) can improve the diagnostic and differential diagnostic accuracy of childhood hepatic tumors.

**Methods:**

A multi-center prospective observational study was performed at nine regional institutions around China. Children with hepatic mass (Group T) were divided into hepatoblastoma group (Group T_HB_) and hemangioendothelioma group (Group T_HE_), children with extrahepatic abdominal mass (Group C). Peripheral blood was collected from each patient prior to surgery or chemotherapy. The area under the curve (AUROC) was used to evaluate the diagnostic efficiency of PIVKA-II and the combined tumor markers with AFP.

**Results:**

The mean levels of PIVKA-II and AFP were both significantly higher in Group T than Group C (*p* = 0.001, *p* < 0.001), in Group T_HB_ than Group T_HE_ (p = 0.018, p = 0.013) and in advanced HB than non-advanced HB (p = 0.001, p = 0.021). For the diagnosis of childhood hepatic tumors, AUROC of PIVKA-II (cut-off value 32.6 mAU/mL) and AFP (cut-off value 120 ng/mL) was 0.867 and 0.857. The differential diagnostic value of PIVKA-II and AFP in hepatoblastoma from hemangioendothelioma was further assessed, AUROC of PIVKA-II (cut-off value 47.1mAU/mL) and AFP (cut-off value 560 ng/mL) was 0.876 and 0.743. The combined markers showed higher AUROC (0.891, 0.895 respectively) than PIVKA-II or AFP alone.

**Conclusions:**

The serum level of PIVKA-II was significantly higher in children with hepatic tumors, especially those with malignant tumors. The combination of PIVKA-II with AFP further increased the diagnostic performance.

**Trial registration:**

Clinical Trials, NCT03645655. Registered 20 August 2018, https://www.clinicaltrials.gov/ct2/show/NCT03645655.

## Introduction

Although hepatic tumors rarely occur during childhood, they are associated with significantly higher morbidity and mortality in affected patients. Hepatoblastoma (HB) is the most common malignant hepatic tumor in children under the age of 3 years [1], and comprise approximately 5% of the total neoplasms of various types occurring in young children [2]. The clinical features of HB are nonspecific but include the presence of an upper abdominal mass, loss of appetite, weight loss, anemia, jaundice, and ascites, all of which can seriously endanger the lives and health of children. Though the overall survival (OS) has improved dramatically during the past 30 years, patients of advanced stage hepatoblastoma still surfing poor outcome [3].

Hemangioendothelioma (HE) is the most common hepatic vascular tumor in infants less than 6 months of age, with a prevalence of 1% [4]. Most patients with HE present with an asymptomatic abdominal mass and hepatomegaly, but these tumors may be associated with high-output cardiac failure due to the presence of arteriovenous shunts within the tumor [5].

Monitoring and early diagnosis play a vital role in the treatment of childhood hepatic tumors. In some clinical practices, ultrasound and contrast-enhanced computed tomography (CECT) are used as the primary modalities for the evaluation of palpable abdominal masses and the screening of hepatic masses [6].

Although alpha-fetoprotein (AFP) has been recognized as a biomarker of hepatic tumors [7], it is not always elevated in all hepatic tumor cases. Elevated AFP levels alone are not sufficient for the diagnosis of hepatic tumors due to the physiological elevation seen in normal infants during the first 8 months [8] and because of their association with other primary tumors.

The protein induced by the vitamin K antagonist-II (PIVKA-II) is also known as des-γ-carboxyprothrombin (DCP) or carboxy prothrombin and is an abnormal form of prothrombin induced by the absence of vitamin K or antagonist-II [9]. Motohara, reported PIVKA-II levels were highly elevated in all three hepatoblastoma patients in 1987; plasma PIVKA-II might be useful as a new marker of hepatoblastoma [10].

Elevation of PIVKA-II, due to an excess production by tumor cells, has been shown to be associated with hepatocellular carcinoma (HCC) [9, 11]. Many studies have demonstrated the clinical value of PIVKA-II for HCC surveillance, and PIVKA-II has been recommended by the guidelines of the Japan Society of Hepatology (JSH) [12].

Theoretically, AFP and PIVKA-II are independently produced by tumors and are not correlated with one another. The diagnostic accuracy was better when using a combination of the biomarkers, AFP and PIVKA-II, compared to each marker alone for detecting HCC and early HCC in cirrhotic patients [13, 14]. Measurement of both PIVKA-II and AFP levels may yield useful information on the treatment response and prognosis in HCC patients [15, 16].

Given their application in HCC, we intend to investigate whether PIVKA-II combined with AFP can also improve the diagnostic and differential diagnostic accuracy of childhood hepatic tumors.

## Materials and methods

### Study design

This is a multicenter prospective observational study sponsored by the Shanghai Children’s Medical Center and joined by eight regional institutions around China, including the Children’s Hospital of Fudan University, the Children’s Hospital of Chongqing Medical University, the Children’s Hospital of Nanjing Medical University, the Qilu Children’s Hospital of Shandong University, the Children’s Hospital Affiliated to Zhengzhou University, the Anhui Provincial Children’s Hospital, the Sun Yat-Sen Memorial Hospital, the Sun Yat-Sen University and the First Affiliated Hospital of Anhui Medical University. The study was conducted in accordance with the Declaration of Helsinki, and all participating centers obtained the relevant Institute Review Board ethics committee approval before patient enrollment. The study was registered in http://register.clinicaltrials.gov as NCT03645655.

### Eligible population

Children (age ≤ 144 months) diagnosed with an abdominal mass firstly in the pediatric general surgery inpatient department from October 1, 2018 to September 30, 2020 were consecutively enrolled in this study. The diagnosis of hepatoblastoma was based on serum biomarkers, contrast-enhanced computed tomography (CECT) and histopathology according to the International Childhood Liver Tumors Strategy Group (SIOPEL) protocols [17]. The diagnosis of hemangioendothelioma was based on a combination of clinical findings and CECT, biopsy was performed if the clinical findings or CECT imaging were atypical [18]. A cavitation ultrasonic surgical aspirator (Soering GmbH) was used for tumor biopsies and resections, which was safe and reliable. Children confirmed to have a hepatic mass were placed in the testing group (Group T) and were further divided into the hepatoblastoma group (Group T_HB_) and the hemangioendothelioma group (Group T_HE_); the other children confirmed to have an extrahepatic abdominal mass were placed in the control group (Group C). Advanced stage hepatoblastoma, including both locally advanced primary tumors (PRETEXT III/IV) as well as metastatic disease [19]. Informed consent was obtained from each child’s legal guardian. Children with extra-abdominal tumors, neoadjuvant chemotherapy history, ongoing vitamin K or warfarin treatment or lacking informed consent were excluded from this study.

### Laboratory measurements

Peripheral blood was collected from each patient prior to any treatment (surgery and/or chemotherapy). Blood samples were centrifuged, and serum was aliquoted and stored at − 80 °C. All serum samples were tested in a single center to decrease the possibility of bias. Serum levels of AFP and PIVKA-II were determined by a chemiluminescence enzyme immunoassay (CLEIA) (ARCHITECH 2000, Abbott Laboratory, US) using an enzyme-linked immunosorbent assay kit (Abbott Laboratory, US) per the manufacturer’s instructions. All samples were analyzed in duplicate.

### Sample size calculation

A sample size calculation was performed using PASS 15.0 (Power Analysis and Sample Size software, NCSS, Kaysville, UT, US) using the log-rank test. The planned sample size was determined after assuming the use of a 2-sided log-rank test with a type I error rate of 0.05 and a statistical power of 90%. A dropout rate of up to 20% was factored into the computations. Ninety-three patients in each group were asked to participate in this study.

### Statistical analysis

Student’s t test (or Wilcoxon test) was used to compare the continuous variables, and the chi-square test (or Fisher’s exact test) was used for the categorical variables. The average tumor marker levels were compared between Group T and Group C and between Group T_HB_ and Group T_HE_. Receiver operating characteristic curves (ROCs) and area under the ROC curve (AUROC) were used to assess the diagnostic and differential diagnostic efficiencies of PIVKA-II, AFP and the combination of the two tumor markers. For patients under 1 year of age (32 cases in Group T_HB_, 22 cases in Group T_HE_ and 11 cases in Group C), the AFP statistical value was adjusted to the test value minus the average normal value according to different months of age [8]. A two-sided p value less than 0.05 was considered statistically significant. All statistical analyses were carried out with SPSS version 20.0 (SPSS Inc., an IBM Company, Chicago, IL, US).

## Results

### Patient characteristics

**Fig. 1:**
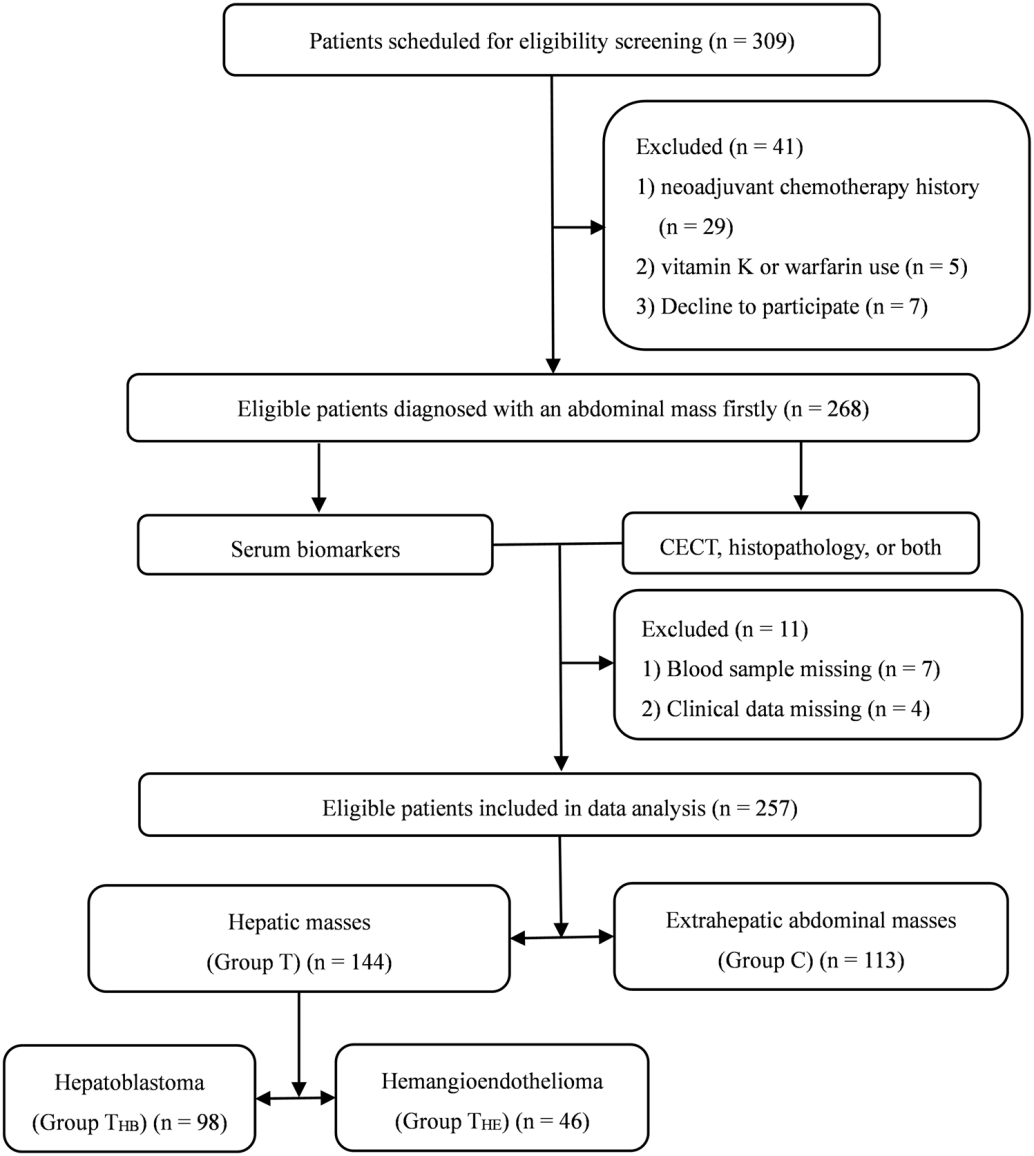
Flowchart of patient enrolment. *CECT* contrast-enhanced computed tomography, *HB* hepatoblastoma, *HE* hemangioendothelioma

A total of 257 eligible patients with available data were enrolled in this study from October 1, 2018, to September 30, 2020 (Fig. 1). Table 1 shows the demographics of the participants. A total of 144 patients (mean age 24.4 ± 28.5 months) were confirmed to have hepatic masses (Group T), 98 patients (mean age 28.4 ± 31.6 months) were diagnosed with hepatoblastoma (Group T_HB_), 46 patients (mean age 16.0 ± 18.2 months) were diagnosed with hemangioendothelioma (Group T_HE_), and the other 113 patients (mean age 35.8 ± 28.9 months) were confirmed to have extrahepatic abdominal masses (Group C). Except for thrombin time (TT, p = 0.003), there were no significant differences in age (p = 0.156), sex (p = 0.159), platelet count (PLT, p = 0.466), prothrombin time (PT, p = 0.078), activated partial thromboplastin time (APTT, p = 0.065), fibrinogen (FIB, p = 0.120), aspartate amino transferase (AST, p = 0.262), alanine amino transferase (ALT, p = 0.442), gamma-glutamyl transpeptidase (GGT, p = 0.924), total protein (TP, p = 0.604), albumin (ALB, p = 0.083) or total bilirubin (TBIL, p = 0.897) between Group T and Group C. Patients in Group THE were younger than Group THB (p = 0.014), the tumor size was smaller (p < 0.001); except for AST (p = 0.035), there were no significant differences in sex (p = 0.075), PLT (p = 0.355), PT (p = 0.069), TT (p = 0.584), APTT (p = 0.340), FIB (p = 0.071), ALT (p = 0.218), GGT (p = 0.779), TP (p = 0.564), ALB (p = 0.378), or TBIL (*p* = 0.092) between Group T_HB_ and Group T_HE_.

**Table 1:** Baseline characteristics of patients

	Group T	Group C	*p*		Group T_HB_	Group T_HE_	*p*
Sample size	144	113			98	46	
Age (Month)	24.4 ± 28.5	35.8 ± 28.9	0.156		28.4 ± 31.6	16.0 ± 18.2	**0.014**
Gender			0.159				0.075
Male, n (%)	79 (54.9)	52 (46.0)			53 (54.1)	26 (56.5)	
Female, n (%)	65 (45.1)	61 (54.0)			45 (45.9)	20 (43.5)	
Tumor size (mm)	95.978 ± 43.219	105.817 ± 76.645	0.195		115.281 ± 35.565	54.854 ± 26.655	** < 0.001**
PLT (× 10^9^/L)	468.958 ± 192.419	487.867 ± 221.883	0.466		479.153 ± 201.199	447.231 ± 72.308	0.355
PT (s)	10.917 ± 1.796	11.297 ± 1.603	0.078		11.103 ± 1.522	10.520 ± 2.240	0.069
TT (s)	17.105 ± 2.740	16.023 ± 3.022	**0.003**		17.191 ± 2.627	16.921 ± 2.988	0.584
APTT (s)	36.297 ± 5.743	37.589 ± 5.283	0.065		36.611 ± 5.577	35.628 ± 6.089	0.340
FIB (g/L)	2.266 ± 0.899	2.427 ± 0.714	0.120		2.173 ± 0.897	2.463 ± 0.878	0.071
AST (UI/L)	52.944 ± 30.352	50.575 ± 35.833	0.262		56.591 ± 31.500	45.174 ± 26.421	**0.035**
ALT (UI/L)	31.458 ± 18.987	30.832 ± 32.638	0.442		32.800 ± 21.471	28.609 ± 11.816	0.218
GGT (UI/L)	31.479 ± 25.966	25.319 ± 45.375	0.924		31.898 ± 21.287	30.587 ± 34.126	0.779
TP (g/L)	63.217 ± 7.428	69.133 ± 8.352	0.604		63.462 ± 6.522	62.694 ± 9.123	0.564
ALB (g/L)	41.522 ± 5.553	42.412 ± 6.390	0.083		41.241 ± 4.783	42.120 ± 6.941	0.378
TBIL (μ mol/L)	17.172 ± 14.139	15.988 ± 22.637	0.897		18.534 ± 14.287	14.272 ± 13.516	0.092

*Group T* hepatic mass group, *Group C* extrahepatic abdominal mass group, *Group THB* hepatoblastoma group, *Group THE* hemangioendothelioma group, *ALB* albumin, *ALT* alanine amino transferase, *APTT* activated partial thromboplastin time, *AST* aspartate amino transferase, *FIB* fibrinogen, *GGT* gamma-glutamyl transpeptidase, *PLT* platelet count, *PT* prothrombin time, *TBIL* total bilirubin, *TP* total protein, *TT* thrombin time. Bold show p < 0.05

### Serum levels of PIVKA-II and AFP

**Fig. 2:**
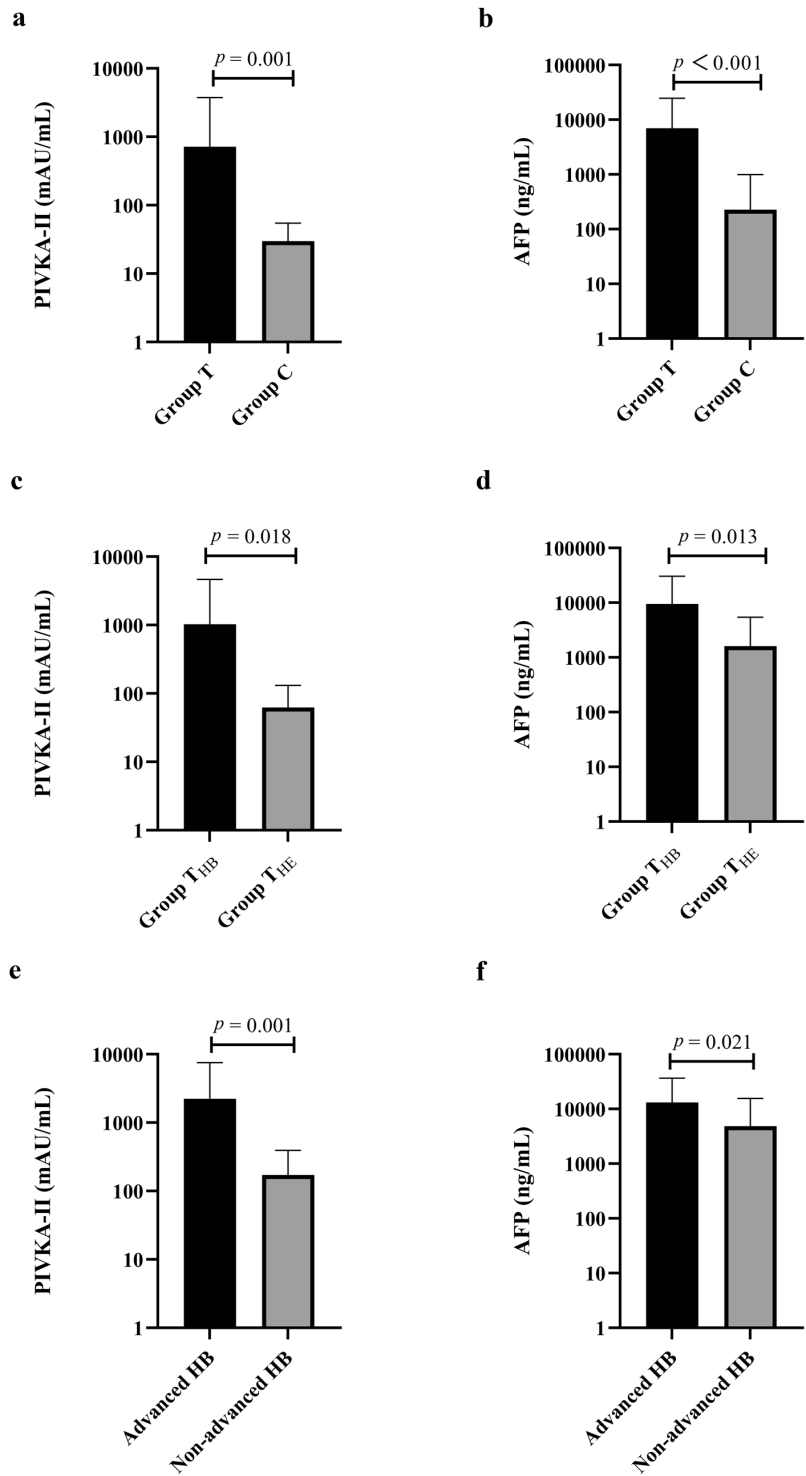
Serum levels of PIVKA-II and AFP. **a**, **b** Serum PIVKA-II and AFP levels in Group T and Group C patients; **c**, **d** Serum PIVKA-II and AFP levels in Group T_HB_ and Group T_HE_ patients; **e**, **f** Serum PIVKA-II and AFP levels in advanced HB group and non-advanced HB group patients. *PIVKA-II* protein induced by vitamin K absence-II, *AFP* alpha-fetoprotein, *Group T* hepatic mass group, *Group C* extrahepatic abdominal mass group, *Group T*_*HB*_ hepatoblastoma group, *Group T*_*HE*_ hemangioendothelioma group

Serum PIVKA-II and AFP levels were compared between the patients in the hepatic mass group and the patients in the control group and between the patients in the hepatoblastoma group and the patients in the hemangioendothelioma group. The mean level of PIVKA-II in Group T was 717.687 ± 3026.936 mAU/mL, which was significantly higher than that of Group C (29.954 ± 24.924 mAU/mL, *p* = 0.001) (Fig. 2a). The mean level of AFP in Group T was significantly higher than that in Group C (6982.617 ± 17,833.972 ng/mL vs 226.368 ± 772.413 ng/mL, *p* < 0.001) (Fig. 2b).

A similar trend was found in serum PIVKA-II and AFP levels between Group T_HB_ and Group T_HE_. Serum levels of PIVKA-II and AFP were both significantly higher in Group T_HB_ than Group T_HE_ (PIVKA-II: 1025.091 ± 3634.021 mAU/mL vs 62.467 ± 68.900 mAU/mL, *p* = 0.018; AFP: 9504.202 ± 21,023.325 ng/mL vs 1610.545 ± 3825.377 ng/mL, *p* = 0.013) (Fig. 2c, d).

In the HB group, Serum levels of PIVKA-II and AFP in patients with advanced HB (n = 75) were significantly higher than those in patients with non-advanced HB (n = 23), PIVKA-II: 2229.376 ± 5300.046 mAU/mL vs 172.413 ± 219.713 mAU/mL, p = 0.001; AFP: 13,082.426 ± 23,507.643 ng/mL vs 4833.033 ± 10,733.654 ng/mL, p = 0.021 (Fig. 2e, f).

### Diagnostic values of PIVKA-II and AFP in childhood hepatic tumor patients

**Fig. 3:**
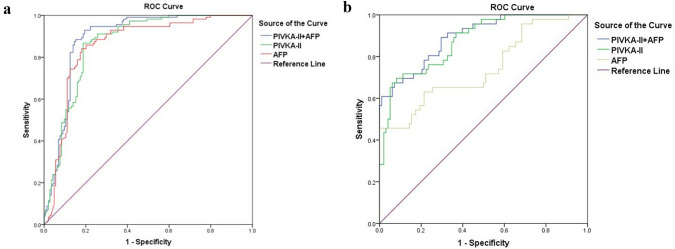
Diagnostic values of PIVKA-II and AFP in childhood hepatic tumor patients. **a** The AUROCs of PIVKA-II, AFP and PIVKA-II + AFP for the diagnosis of hepatic tumors were 0.867, 0.857 and 0.891, respectively. **b** The AUROCs of PIVKA-II, AFP and PIVKA-II + AFP to differentiate hepatoblastoma from hemangioendothelioma patients were 0.876, 0.743 and 0.895, respectively. *PIVKA-II* protein induced by vitamin K absence-II, *AFP* alpha-fetoprotein

To evaluate the diagnostic values of PIVKA-II and AFP in childhood hepatic tumor patients, ROC curves were plotted to identify the cutoff values that would best differentiate hepatic tumor patients from controls. The area under the ROC curve (AUROC) of PIVKA-II was 0.867 (95% CI 0.822–0.911, *p* < 0.001), and the AUROC of AFP was 0.857 (95% CI 0.808–0.906, *p* < 0.001). The optimal cutoff value of PIVKA-II was 32.6 mAU/mL, the sensitivity was 86.7%, and the specificity was 81.3%. The optimal cutoff value of AFP was 120 ng/mL, the sensitivity was 84.1%, and the specificity was 81.9%. Serum levels of PIVKA-II and AFP were then combined to obtain a new marker for childhood hepatic tumor diagnosis. ROC analysis showed that PIVKA-II + AFP further increased the diagnostic efficiency. The AUROC was 0.891 (95% CI 0.850–0.933, p < 0.001), higher than that of PIVKA-II (p = 0.029) or AFP (p = 0.031) alone. The combined sensitivity and specificity were 88.5% and 84.7%, respectively (Fig. 3a).

**Fig. 4:**
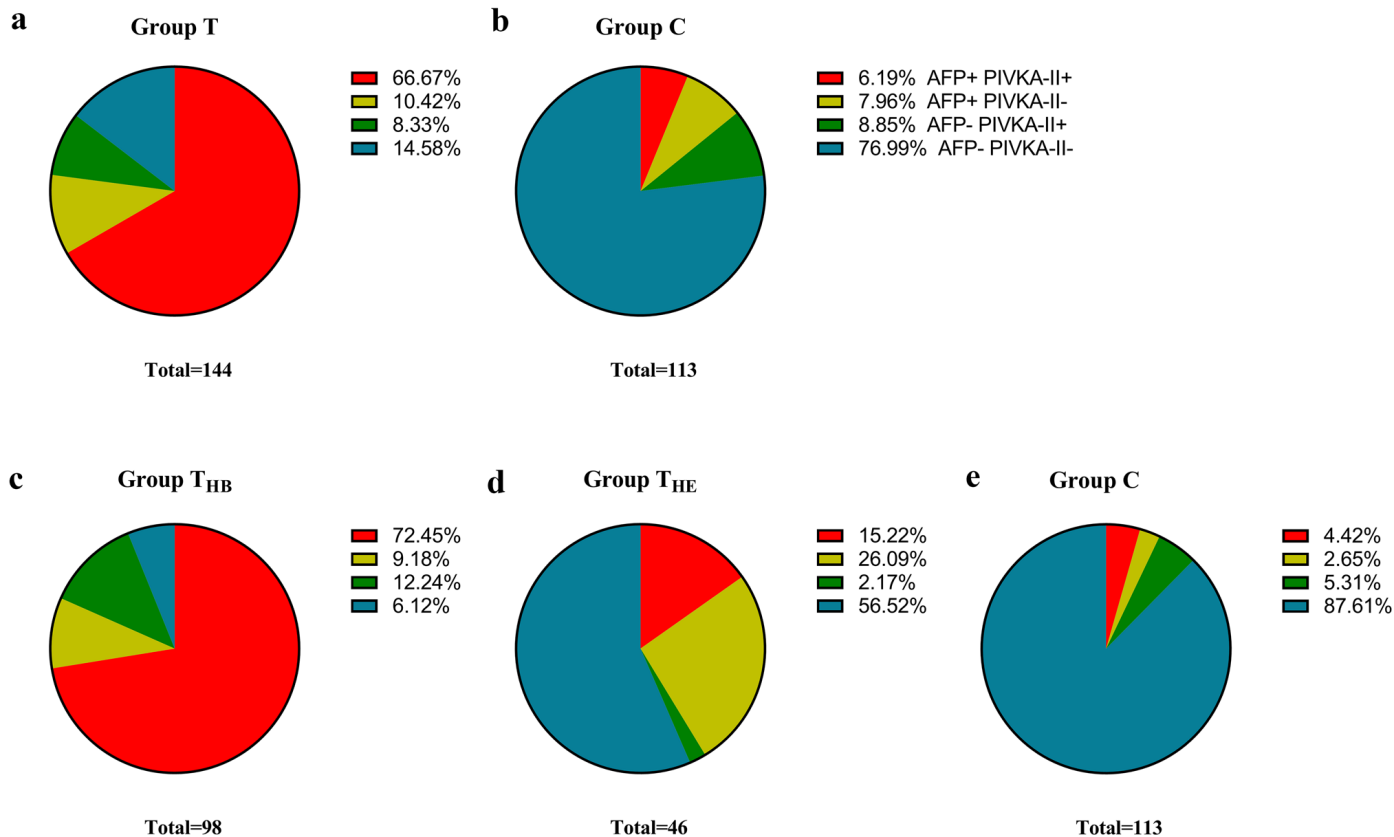
Pie charts of patients above and below the cutoff values of biomarkers. **a**, **b** The percentages of patients in Group T and Group C, PIVKA-II cutoff value = 32.6 mAU/mL, AFP cutoff value = 120 ng/mL; **c**–**e** The percentages of patients in Group T_HB_, Group T_HE_ and Group C_,_ PIVKA-II cutoff value = 47.1 mAU/mL, AFP cutoff value = 560 ng/mL

The percentages of patients above and below the cutoff values of biomarkers in Group T and Group C were shown in Fig. 4a and b. The proportion of patients with combined AFP + and/or PIVKA + in Group T were higher than that in Group C (85.42% vs 23.01%, p < 0.001).

### Differential diagnostic values of PIVKA-II and AFP in hepatoblastoma patients

To further assess the diagnostic value of PIVKA-II and AFP levels in differentiating hepatoblastoma patients from hemangioendothelioma patients, another ROC curve was constructed. The AUROC of PIVKA-II was 0.876 (95% CI 0.818–0.934, *p* < 0.001), and the AUROC of AFP was 0.743 (95% CI 0.651–0.835, *p* < 0.001). The optimal cutoff value of PIVKA-II was 47.1 mAU/mL, sensitivity was 71.7% and specificity was 88.7%. The optimal cutoff value of AFP was 560 ng/mL, sensitivity was 63.0% and specificity was 78.6%. ROC analysis showed that PIVKA-II + AFP further increased the differential diagnostic efficiency. The AUROC was 0.895 (95% CI 0.841–0.948, p < 0.001), which was higher than that of PIVKA-II (p = 0.657) or AFP (p < 0.001) alone. The combined sensitivity and specificity were 72.7% and 91.8% (Fig. 3b).

The percentages of patients above and below the cutoff values of biomarkers in Group THB and Group THE were shown in Fig. 4c, d and e. The proportion of patients with combined AFP + and/or PIVKA + in Group THB were higher than that in Group THE (93.88% vs 43.48%, p < 0.001) and Group C (93.88% vs 12.39%, p < 0.001).

## Discussion

Regular monitoring and an early diagnosis of childhood tumors can improve the clinical course and treatment response, which ultimately improves long-term outcomes [20]. The blood tumor markers that are described in this study can be considered good indicators and can provide an acceptable diagnostic accuracy and are convenient and cost-effective.

The results of this study showed that hepatic tumor patients had significantly higher serum levels of PIVKA-II and AFP than extrahepatic abdominal tumor patients.

Prothrombin glutamate carboxylation in the liver gives rise to normal prothrombin, which contains 10-carboxylic glutamate residues. The process depends on the presence of vitamin K. In pathological states when vitamin K is too low or in the presence of a vitamin K-dependent antagonist of carboxylase, the insufficient carboxylation of glutamic acid results in the production of PIVKA-II [21].

Motohara et al. reported that vitamin K treatment in two hepatoblastoma patients resulted in only a moderate reduction in PIVKA-II levels. An immunohistochemical study of liver tissue showed the presence of PIVKA-II in hepatoblastoma cells [10]. Maha et al. reported similar outcomes; after vitamin K administration, PIVKA-II levels decreased in both the chronic hepatitis group (*p* = 0.022) and the cirrhosis group (*p* = 0.024) but not in the HCC group (*p* = 0.187) [22]. These findings suggested that the elevation of PIVKA-II in patients with liver tumors was not due to deficiency in the nutrient vitamin K but due to the overproduction of PIVKA-II in tumor cells.

The subgroup analysis showed that PIVKA-II and AFP levels in patients with malignant hepatoblastoma were higher than those with the benign hemangioendothelioma, in our study. Furthermore, PIVKA-II and AFP levels were higher in advanced stage HB patients than those in non-advanced stage HB patients.

Imamura et al. reported that serum PIVKA-II levels were significantly elevated in patients with more aggressive tumor characteristics [23]. Recently, many studies have demonstrated that elevated serum PIVKA-II is related to larger tumor size, more frequent vascular invasion, more intrahepatic metastasis, and recurrence after treatment [24].

A couple of previous studies reported that the optimal cutoff value of serum PIVKA-II for HCC diagnosis was estimated to range from 30 to 42 mAU/mL [25]. The ROC curve analysis showed that the optimal cutoff value of PIVKA-II for the diagnosis of childhood hepatic tumors was 32.6 mAU/mL and that for differentiating hepatoblastoma from hemangioendothelioma was 47.1 mAU/mL. The cutoff values of serum PIVKA-II for the diagnosis of hepatic tumors in children and adults were similar and were not affected by age.

In previous studies, the sensitivity of PIVKA-II in the diagnosis of HCC was 51.0–77%, the specificity was 67.8–91.2%, and the AUROC was 0.701–0.854, all of which were higher than the sensitivity, specificity, and the AUROC of AFP [16, 26]. In our study, the sensitivity, specificity and AUROC of PIVKA-II in the diagnosis of childhood hepatic tumors and in the differentiation of hepatoblastoma from benign hepatic tumors were all higher than AFP. PIVKA-II is a good marker with good sensitivity and specificity in the diagnosis of hepatic tumors in both children and adults.

Serum PIVKAII and AFP are produced through different mechanisms. AFP secretion in HCC results from a re-expression of a fetal antigen in the tumor, and PIVKA-II results from an independently acquired posttranslational defect in protein processing [27]. Therefore, the two markers are independent from each other in the diagnosis of hepatic tumors [28]. A few studies reported that PIVKA-II combined with AFP had great advantages as a biomarker for HCC screening [29]. The maximum AUROC was 0.846, which was higher than that of PIVKA-II or AFP alone [30]. We further evaluated the diagnostic performance of the combination of the two markers. The results showed that the combination of PIVKA-II and AFP further increase the efficiency for the diagnosis of childhood hepatic tumors (AUROC = 0.891) and for the differentiation of hepatoblastoma from benign hepatic tumors (AUROC = 0.895). Our study was broadly consistent with these findings.

This study has a few limitations. The first, lack of external validation. Although this is a multicenter clinical study, we only included hospitals in about a quarter of China’s provinces and restricted to children under 12 years of age with abdominal mass. Thus, these findings may not be generalizable to pediatric patients in other parts of China. The second, the values of PIVKA-II combined with AFP in post-treatment surveillance and clinical outcome prognosis of childhood hepatic tumors are lacking, which require further research in the future.

## Conclusion

This study demonstrated that the serum level of PIVKA-II was significantly higher in childhood patients with hepatic tumors, especially in those with malignant tumors. As a biomarker, PIVKA-II had superior sensitivity and specificity in the diagnosis of hepatic tumors, and its cutoff value was not affected by age. The combination of PIVKA-II with AFP further increased the diagnostic performance. Therefore, serum PIVKA-II combined with AFP levels may be considered a screening marker for the clinical diagnosis of childhood hepatic tumors.

## Data Availability

All data generated or analyzed during this study are included in this published article.

## Abbreviations

AFP: α-Fetoprotein
ALB: Albumin
ALT: Alanine amino transferase
APTT: Activated partial thromboplastin time
AST: Aspartate amino transferase
AUROC: Area under the curve
CECT: Contrast-enhanced computed tomography
CLEIA: Chemiluminescence enzyme immunoassay
DCP: Des-γ-carboxyprothrombin
FIB: Fibrinogen
GGT: Gamma-glutamyl transpeptidase
HB: Hepatoblastoma
HCC: Hepatocellular carcinoma
HE: Hemangioendothelioma
JSH: Japan Society of Hepatology
PIVKA-II: Protein induced by vitamin K antagonist-II
PLT: Platelet count
PT: Prothrombin time
ROC: Receiver operating characteristics
SIOPEL: International Childhood Liver Tumors Strategy Group
TBIL: Total bilirubin
TP: Total protein
TT: Thrombin time
